# A decade of change: evolving epidemiology of invasive pulmonary mold infections in hematologic malignancy patients from a longitudinal infection control surveillance program

**DOI:** 10.1017/ice.2026.10456

**Published:** 2026-06

**Authors:** Guy Handley, Jane Powell, Dimitrios P. Kontoyiannis, Adina Feldman, Micah Bhatti, Roy F. Chemaly, Amy Spallone

**Affiliations:** 1 Department of Infectious Diseases, Infection Control and Employee Health, The University of Texas MD Anderson Cancer Center, Houston, TX, USA; 2 Department of Infection Control, Chief Quality Office, The University of Texas MD Anderson Cancer Center, Houston, TX, USA; 3 Department of Laboratory Medicine, The University of Texas MD Anderson Cancer Center, Houston, TX, USA

## Abstract

Epidemiologic trends in pulmonary mold infections were assessed. Of 227 infections, 24 were considered late-onset (>14 days) and Aspergillus less common (58.3% vs 78.3%; P = .030). For late-onset cases, Aspergillus was dramatically less frequent from 2020–2025 compared to 2014–2019 (11.1% vs 86.7%, P < .001), while Fusarium spp. increased (44.4% vs 0%, P = .012).

Invasive fungal infections carry high morbidity and mortality in patients living with cancer. While most cases are sporadic and likely community-acquired, nosocomial transmission may occur, particularly during periods of construction or bioaerosol-generating activities.^1–3^ The Centers for Disease Control and Prevention (CDC) and the Infectious Diseases Society of America (IDSA) do not recommend routine bioaerosol sampling due to a paucity of data demonstrating clinical benefit.^4,5^ Nevertheless, they note that sampling may be indicated in the setting of an outbreak or as part of institutional quality assurance mechanisms. Additionally, IDSA recommends that centers providing care for patients with leukemia or transplantation should perform regular surveillance of invasive mold infections to evaluate for potential outbreaks.^5^ However, specific recommendations for how these processes should be structured and implemented are lacking, and practices vary across institutions. Invasive mold infections are routinely monitored at our National Cancer Institute (NCI)-designated Comprehensive Cancer Center as part of standard practice in a robust Infection Prevention and Control (IPC) program. In this study, we reviewed cases of invasive mold pulmonary infections identified during epidemiologic surveillance to evaluate changes in epidemiologic patterns over time.

## Methods

We conducted a retrospective, longitudinal surveillance study of invasive pulmonary mold infections among patients with hematologic malignancies admitted from January 1, 2014, through December 31, 2025. The study was performed as part of an institutional IPC surveillance program designed to detect healthcare-associated fungal infections and monitor epidemiologic trends.

Case identification was performed using a standardized daily report generated from the electronic medical record. In collaboration with the clinical microbiology laboratory, laboratory tests, and specimen labels were defined to ensure comprehensive capture of all positive cultures. Cases with invasive pulmonary mold infections in patients with hematologic malignancy were then identified and reviewed.

All microbiologic cultures isolating *Aspergillus* spp., *Fusarium* spp., *Scedosporium* spp., and Mucorales are routinely monitored by our IPC program. Data were collected on admission date, collection date, service line, culture source, and organism(s) to monitor potential nosocomial outbreaks. A potential nosocomial outbreak was defined as two or more cases with the same organism, not present at the time of admission, linked in time, and geographic location within the institution. Cases were assigned an epidemiologic definition by a certified Infection Preventionist based on the timing of signs or symptoms relative to admission. A community-acquired/early-onset case occurred ≤ 14 days after admission, while a possible nosocomial/late-onset case occurred >14 days following admission. Infections versus colonization/contamination was determined by the Infectious Diseases physician consultant. In cases of indeterminate epidemiologic designation, the case was independently reviewed by the Chief Infection Control Officer for final categorization (Supplemental Figure 1). Epidemiologic trends were then compared from 2014–2019 to 2020–2025.

## Results

**Figure 1.**
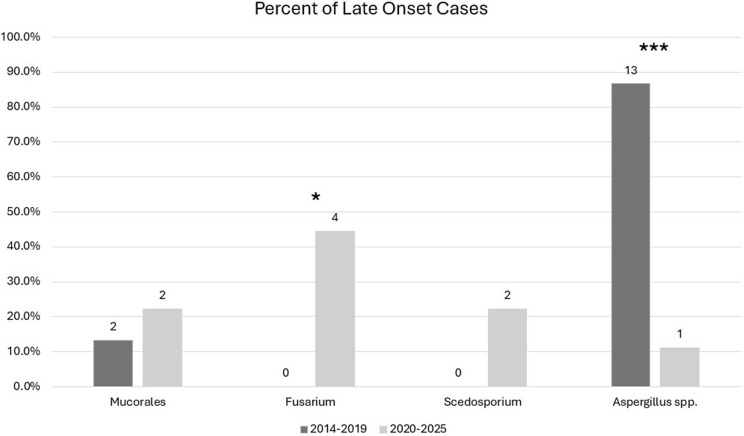
Figure 1 long description. Late-onset isolates 2014–2019 versus 2020–2025. *<0.05, **<0.01, ***<0.001.

Overall, 599 cultures were evaluated from respiratory sources. Of these, 307 occurred in adult patients on hematologic service lines, and 252 (82.1%) were considered infections. Overall 55 cases (28 from 2014–2019 to 27 from 2020–2025) were considered colonization/contamination and excluded. Twenty-five instances of multiple or duplicate cultures collected from the same patients were further excluded. Two hundred and twenty-seven cases in 224 unique patients met the EORTC/MSGERC consensus definitions for probable invasive pulmonary mold disease and were included in the analysis.^6^ Three patients had separate infections separated at least six months apart with different organisms that were also included (Supplemental Figure 2). Overall, 24 cases (10.6%) were classified as late onset/possible nosocomial (Table 1). Multiple molds were isolated in 18 (7.9%) cases; however, only one of these was in a late-onset case. *Aspergillus* spp. were more common from 2014–2019 than 2020–2025 (84.0% vs 70.1%; *P* = .014). This was particularly evident in the late-onset Aspergillus species cohort (Figure 1) from 2014–2019 compared to 2020–2025 (86.7% vs 11.1%, *P* < .001). Additionally, a rise in *Fusarium* spp. was observed during 2020–2025 compared with 2014–2019 (11.8% vs 2.0%, *P* = .005), particularly in the late-onset cohorts (44.4% vs 0%, *P* = .012). No nosocomial outbreaks were detected during the study period.

**Table 1.** Characteristics of invasive molds monitored Table 1 long description.

Organism	Total n = 227^a^	Early onset n = 203	Late onset n = 24	*P*-value	2014–2019 n = 100	2020–2025 n = 127	*P*-value
*Aspergillus* species	173 (76.2)	159 (78.3)	14 (58.3)	.030	84 (84.0)	89 (70.1)	.014
*A. fumigatus*	53 (23.3)	47 (23.2)	6 (25.0)	.840	33 (33.0)	20 (15.7)	.002
*A. terreus*	37 (16.3)	36 (17.7)	1 (4.2)	.068	19 (19.0)	18 (14.2)	.328
*A. flavus*	17 (7.5)	16 (7.9)	1 (4.2)	.442	10 (10.0)	7 (5.5)	.202
*A. niger*	35 (15.4)	32 (15.8)	3 (12.5)	.475	12 (12.0)	23 (18.1)	.206
*Aspergillus*, other	41 (18.1)	38 (18.7)	3 (12.5)	.335	14 (14.0)	27 (21.3)	.158
Mucorales spp.	29 (12.8)	25 (12.3)	4 (16.7)	.367	10 (10.0)	19 (15.0)	.266
*Fusarium* spp.	17 (7.5)	13 (6.4)	4 (16.7)	.089	2 (2.0)	15 (11.8)	.005
*Scedosporium* spp.	12 (5.3)	10 (4.9)	2 (8.3)	.369	4 (4.0)	8 (6.3)	.442

a Multiple molds isolated in 18 (7.9%) of patients.

## Discussion

During the study period, *Aspergillus* spp. remained the most commonly isolated organism in line with historical studies.^7,8^ Similar to more expansive autopsy-based studies, the proportion of *Aspergillus* spp. decreased in later years.^9^ Interestingly, epidemiology differed substantially in patients with late-onset invasive pulmonary mold disease, and while overall *Aspergillus* spp. comprised a majority of late-onset cases, in the 2020–2025 cohort, they made up only 11.1% of cases, while there was a substantial increase in *Fusarium*, which comprised 44.4% of cases. Within the *Aspergillus* spp., *A. fumigatus* incidence decreased from 2020–2025 compared to 2014–2019 (33% vs 15.7%, *P* = .002) as other species increased. While a clear definition of nosocomial mold infection is lacking, we evaluated cases using a 14-day cutoff to better identify epidemiologic trends and potential nosocomial cases. The standard definition of hospital-acquired infection (HAI), with onset 48 hours after hospitalization and absent on admission, is clearly insufficient, as the incubation period of *Aspergillus* has been proposed to be up to 100 days; other proposed cutoffs have included 7 or 10 days.^3^ Patient factors, inoculum size, or additional community and healthcare system exposures likely contribute further risks of nosocomial fungal infection acquisition, which are more difficult to capture in a standard HAI definition. Additionally patients with hematologic malignancy typically have frequent intermittent healthcare system exposures in both inpatient and outpatient settings further complicating the development of standard definitions. However, epidemiologic knowledge based on this 14-day cutoff provides helpful guidance to clinicians and IPC teams alike, particularly those caring for patients with expected prolonged hospitalization, such as those with leukemia or in transplant centers.

Guidelines from the IDSA and the CDC for Aspergillosis underscore protected environmental precautions such as high efficiency particulate air filtration, laminar flow, positive pressure rooms, and avoidance of proximity to construction to reduce the risk of infections for patients.^2,4,5^ While our surveillance program did not detect any possible nosocomial outbreaks, it is important to note the challenges to distinguish nosocomial from community-acquired invasive Aspergillosis.^4,5^ It is well-known that hospitals may house reservoirs of molds such as *Aspergillus*, and in particular, construction work can lead to increased airborne spores, but the organism is common in the external environment as well.^1–3^ While IDSA recommends surveillance for centers providing care for patients with leukemia or transplantation, specific guidance or structure beyond monitoring for an increased rate over baseline or in atypical patient populations is lacking. Lastly, IPC programs are challenged by clinical cases that frequently lack positive microbiologic cultures and do not meet the stricter EORTC/MSGERC consensus definitions for probable invasive mold pneumonia.

Our trend toward non-*Aspergillus* molds, particularly in late-onset infections from 2020–2025, accounting for 88.9% of cases, is striking. The reasons for these trends are likely multifactorial. This may include changes in antifungal prophylaxis, antimicrobial prescribing practices, patient populations, the COVID-19 pandemic and associated IPC practices, novel antineoplastic therapeutics, but are beyond the scope of IPC surveillance and require further study and examination. No substantial changes regarding IPC procedures for invasive mold infection surveillance or prevention, major construction projects within the wards for these patient populations, or internal laboratory based microbiologic diagnostic techniques occurred during this study period.

From a clinical standpoint importantly, these non-*Aspergillus* molds carry high mortality rates, increased resistance to antifungal agents, and risk of dissemination to distant sites.^10^ Standardized antifungal susceptibility testing recommendations or established mean inhibitory concentration breakpoints to predict clinical cure are unclear. Treatment regimens vary, and there is a lack of clinical data demonstrating optimal management of these organisms.^10^ While outbreaks with these organisms have been described, they require enhanced scrutiny and study to better determine at-risk patients or modifiable factors to prevent infection.^1^ These deficiencies and the increasing incidence over time of these organisms provide challenges to clinicians and IPC programs at institutions caring for vulnerable patients.

## Supporting information

10.1017/ice.2026.10456.sm001 Handley et al. supplementary material Handley et al. supplementary material

## Supplementary material

The supplementary material for this article can be found at https://doi.org/10.1017/ice.2026.10456.

## Figure 1 Long description

The bar graph titled Percent of Late Onset Cases compares the percentage of late-onset cases of different fungal infections between two time periods, 2014-2019 and 2020-2025. The x-axis lists four types of fungal infections: Mucorales, Fusarium, Scedosporium, and Aspergillus species. The y-axis represents the percentage of late-onset cases, ranging from 0 to 100 percent. Each infection type has two bars: one for the period 2014-2019 and one for 2020-2025. The bars for 2014-2019 are in dark gray, while the bars for 2020-2025 are in light gray. For Mucorales, the percentage is 2 percent for both periods. For Fusarium, the percentage is 0 percent for 2014-2019 and 4 percent for 2020-2025. For Scedosporium, the percentage is 0 percent for 2014-2019 and 2 percent for 2020-2025. For Aspergillus species, the percentage is 13 percent for 2014-2019 and 1 percent for 2020-2025. The graph includes annotations indicating statistical significance: an asterisk for Fusarium and three asterisks for Aspergillus species. All values are approximated.

Navigate back to Figure 1.

## Table 1 Long description

The table presents data on the characteristics of invasive molds monitored, focusing on different organisms and their occurrence over two time periods (2014-2019 and 2020-2025) and onset types (early and late). The table has 8 rows and 7 columns. The columns are labeled Organism, Total n = 227, Early onset n = 203, Late onset n = 24, P-value, 2014-2019 n = 100, 2020-2025 n = 127, and P-value. The rows list different organisms such as Aspergillus species, A. fumigatus, A. terreus, A. flavus, A. niger, Aspergillus other, Mucorales spp., Fusarium spp., and Scedosporium spp. The table shows the number and percentage of cases for each organism across different categories. Notable trends include a higher percentage of Aspergillus species in the early onset and 2014-2019 periods, and a rise in Fusarium spp. during 2020-2025.

Navigate back to Table 1.
