# Supplementary material for: A decade of change: evolving epidemiology of invasive pulmonary mold infections in hematologic malignancy patients from a longitudinal infection control surveillance program
Source: Infect Control Hosp Epidemiol. 2026 Apr 21;47(6):630–2. doi: 10.1017/ice.2026.10456 (PMC13216806; doi:10.1017/ice.2026.10456)
Supplement: Handley et al. supplementary material [file S0899823X26104565sup001.docx]

Supplemental Figures

Supplemental Figure 1: Invasive mold infection surveillance workflow at an NCI-Designated Comprehensive Cancer Center


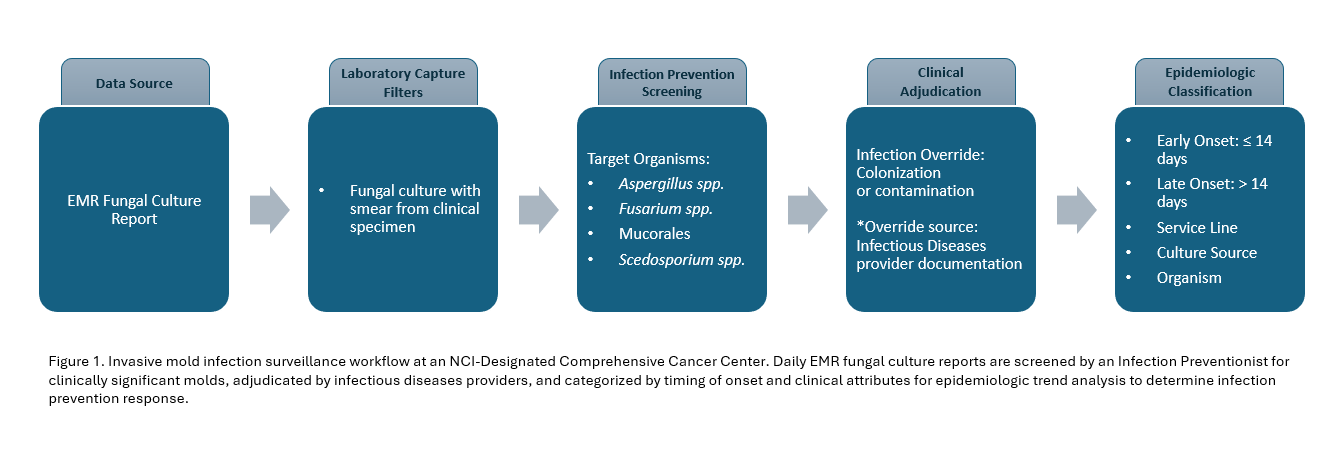


Supplemental Figure 2: Case Selection for Cases Included in Analysis

Positive Mold Cultures Isolated from Respiratory Sources n=599

Excluded Cultures in Patients from Non-Hematology Service Lines n=292

Cases Meeting EORTC/MSGERC Definitions Included in Analysis n=227

Excluded Cultures Deemed Colonization or Contamination n=55

Excluded Duplicate or Multiple Cultures from Identical Patient n=25
